# Supplementary material for: Content-rich biological network constructed by mining PubMed abstracts
Source: BMC Bioinformatics. 2004 Oct 8;5:147. doi: 10.1186/1471-2105-5-147 (PMC528731; doi:10.1186/1471-2105-5-147)
Supplement: Additional File 2 — The original results of the above study (non-essential files are deleted to keep the file size under the limit set by BMC bioinformatics). [file 1471-2105-5-147-S2.bz2 › chilibotAdditionalFile2/dip10/294ID9309223E1126/html/NRDA_NRDB.html]

 


 **NRDA** and **NRDB** 
  
Found 1800 abstracts in PubMed, retrieved 10.  
 

 What does Google say? 
 PDF only 
| .edu only 

---

**Interactive relationship** (e.g. stimulation, inhibition, etc)

**Non-interactive relationship** (e.g. studied together, co-existance, homology, etc.)

- An assay of nucleotide permeable cells for ribonucleotide reductase  [ **NRDB** ]   [ **NRDA** ]  activity using 3H CDP as substrate revealed a pre early and complete decline of DNA precursor biosynthesis not found in the untreated control.  Ref: 12882290 Biotechnol Lett, 2003
- The small subunit of Escherichia coli ribonucleotide reductase  [ **NRDB** ]   [ **NRDA** ]  R2 is a homodimeric betabeta protein, in which each beta peptide contains a diiron cluster composed of two inequivalent iron sites.  Ref: 12862469 J Am Chem Soc, 2003
- p53R2, which is regulated by tumor suppressor p53, is a small subunit of ribonucleotide reductase  [ **NRDB** ]   [ **NRDA** ] .  Ref: 12858174 Nat GenetNat Genet, 2003
- Furthermore, overexpression of suc22, a ribonucleotide reductase  [ **NRDB** ]   [ **NRDA** ]  gene, fully complemented the sporulation defect of cdt2 mutants and alleviated their growth defect at 19 degrees.  Ref: 12871901 Genetics, 2003
- Hydroxyurea, a known S phase specific cytotoxic ribonucleotide reductase  [ **NRDB** ]   [ **NRDA** ]  RR inhibitor, is an effective agent for HbF induction in patients with SCA, but the mechanisms by which hydroxyurea induces HbF in vivo have not been elucidated.Materials and Methods.  Ref: 12842703 Exp Hematol< MedlineTA>Exp Hematol, 2003
- ribonucleotide reductase  [ **NRDB** ]   [ **NRDA** ]  intermediate X.  Ref: 12859213 Acc Chem Res, 2003
- In fission yeast, the COP9 signalosome is required to activate ribonucleotide reductase  [ **NRDB** ]   [ **NRDA** ]  for DNA synthesis.  Ref: 12867051 Curr Biol, 2003
- Mechanistic Implications for the Formation of the Diiron Cluster in Ribonucleotide Reductase  [ **NRDB** ]   [ **NRDA** ]  Provided by Quantitative EPR Spectroscopy.  Ref: 12862469 J Am Chem Soc, 2003
- First, after immunoprecipitation of MHC class I from NXS2 cells, peptides were eluted and examined in tandem MS analysis which lead to the identification of three novel natural MHC class I peptide ligands, TEALPVKLI from ribonucleotide reductase  [ **NRDB** ]   [ **NRDA** ]  M2, NEYIMSLI from Ser Thr protein phosphatase 2A and FEMVSTLI with unknown origin.  Ref: 12880984 Cancer Lett, 2003
- Arrest of cell cycle by inhibition of ribonucleotide reductase  [ **NRDB** ]   [ **NRDA** ]  induces accumulation of NAD by Mn2 supplemented growth of Corynebacterium ammoniagenes.  Ref: 12882290 Biotechnol Lett, 2003
- The most interesting result of analysis of B12 elements is that B12 independent isozymes of the methionine synthase and ribonucleotide reductase  [ **NRDB** ]   [ **NRDA** ]  are regulated by B12 elements in bacteria that have both B12 dependent and B12 independent isozymes.  Ref: 12869542 J Biol ChemJ Biol Chem, 2003
- A simple model is presented to discuss the metabolic regulation of the new process based on the presence of a manganese ribonucleotide reductase  [ **NRDB** ]   [ **NRDA** ]  Mn RNR in the producing strain.  Ref: 12882290 Biotechnol Lett, 2003
- The NO induced dopaminergic cell loss causing Parkinson s disease PD has been postulated to include the following an inhibition of cytochrome oxidase, ribonucleotide reductase  [ **NRDB** ]   [ **NRDA** ] , mitochondrial complexes I, II, and IV in the respiratory chain, superoxide dismutase, glyceraldehyde 3 phosphate dehydrogenase.  Ref: 12880486 Antioxid Redox Signal, 2003
- This is mediated via the ubiquitin ligase Pcu4, activation of which leads to degradation of the scaffold protein Spd1, which anchors the small ribonucleotide reductase  [ **NRDB** ]   [ **NRDA** ]  subunit in the nucleus away from the large subunit in the cytoplasm.  Ref: 12867051 Curr Biol, 2003
